# Factors Associated with First-Pass Success in Pediatric Intubation in the Emergency Department

**DOI:** 10.5811/westjem.2016.1.28685

**Published:** 2016-03-02

**Authors:** Tadahiro Goto, Koichiro Gibo, Yusuke Hagiwara, Masashi Okubo, David F.M. Brown, Calvin A. Brown, Kohei Hasegawa

**Affiliations:** * Massachusetts General Hospital, Department of Emergency Medicine, Boston, Massachusetts; † Okinawa Prefectural Chubu Hospital, Department of Emergency Medicine, Okinawa, Japan; ‡ Tokyo Metropolitan Children’s Medical Center, Division of Paediatric Emergency Medicine, Department of Paediatric Emergency and Critical Care Medicine, Tokyo, Japan; § Mayo Clinic, Department of Emergency Medicine, Rochester, Minnesota; ¶ Brigham and Women’s Hospital, Department of Emergency Medicine, Boston, Massachusetts

## Abstract

**Introduction:**

The objective of this study was to investigate the factors associated with first-pass success in pediatric intubation in the emergency department (ED).

**Methods:**

We analyzed the data from two multicenter prospective studies of ED intubation in 17 EDs between April 2010 and September 2014. The studies prospectively measured patient’s age, sex, principal indication for intubation, methods (e.g., rapid sequence intubation [RSI]), devices, and intubator’s level of training and specialty. To evaluate independent predictors of first-pass success, we fit logistic regression model with generalized estimating equations. In the sensitivity analysis, we repeated the analysis in children <10 years.

**Results:**

A total of 293 children aged ≤18 years who underwent ED intubation were eligible for the analysis. The overall first-pass success rate was 60% (95%CI [54%–66%]). In the multivariable model, age ≥10 years (adjusted odds ratio [aOR], 2.45; 95% CI [1.23–4.87]), use of RSI (aOR, 2.17; 95% CI [1.31–3.57]), and intubation attempt by an emergency physician (aOR, 3.21; 95% CI [1.78–5.83]) were significantly associated with a higher chance of first-pass success. Likewise, in the sensitivity analysis, the use of RSI (aOR, 3.05; 95% CI [1.63–5.70]), and intubation attempt by an emergency physician (aOR, 4.08; 95% CI [1.92–8.63]) were significantly associated with a higher chance of first-pass success.

**Conclusion:**

Based on two large multicenter prospective studies of ED airway management, we found that older age, use of RSI, and intubation by emergency physicians were the independent predictors of a higher chance of first-pass success in children. Our findings should facilitate investigations to develop optimal airway management strategies in critically-ill children in the ED.

## INTRODUCTION

Successful airway management is a critical intervention to stabilize and resuscitate severely-ill and injured children in the emergency department (ED). The importance of first-pass success – successful intubation on the first attempt – has been emphasized as the goal of emergency intubation because studies have demonstrated associations of repeated intubation attempts with higher complication rates.^1^,^2^ Children are known to have a limited physiologic reserve.^3^ Therefore, identifying factors associated with first-pass success in this vulnerable population is essential. A few small studies have reported that the intubator’s specialty and the use of rapid sequence intubation (RSI) were the factors associated with first-pass success.^4^,^5^ However, in contrast to a study from North America that demonstrated the use of RSI was associated with a higher first-pass success,^5^ another study from South Korea found no association between the use of RSI and first-pass success rate.^4^ Therefore, despite the apparent clinical importance, predictors of first-pass success among children in the ED remain largely unknown. To address this knowledge gap in the literature, we analyzed the data from two large multicenter studies of emergency airway management to investigate the predictors of first-pass success in children.

## METHODS

### Study Design and Settings

We conducted a secondary analysis of the data from the Japanese Emergency Airway Network (JEAN) -1 and -2 studies that are designed to characterize current airway management in the EDs across Japan. The study setting, methods of data collection, and measured variables of these studies have been reported elsewhere.^2^,^6^–^13^ Briefly, the JEAN is a consortium of 17 academic and community EDs from different regions across Japan. These EDs had a median of 30,000 patient visits in the ED per year (range, 4,200–67,000), and pediatric patients were treated in all EDs. All EDs of JEAN-1 and JEAN-2 participating centers were staffed with emergency physicians (EP). The institutional review board at each participating institution approved the study with a waiver of informed consent.

### Selection of Participants

The JEAN studies collected information on all patients who underwent intubation attempts in the ED from April 2010 through September 2014. Among these patients, children aged ≤18 years were included in the present study. We excluded children with the use of cricothyroidotomy, tracheostomy, or nasotracheal intubation on the first intubation attempt.^4^

### Data Collection and Processing

After each intubation encounter in the ED, the intubator completed a standardized data collection form.^9^,^14^ Measured variables were age, sex, principal indication for intubation, methods of intubation, all medications used to facilitate intubation, intubation devices, intubator’s level of training and specialty, intubation success or failure, and intubation-associated adverse events.^2^,^7^–^12^

### Statistical Analyses

The outcome measure of interest was success on the first intubation attempt (first-pass success). An attempt was successful if it resulted in a tracheal tube being placed through the vocal cords, with confirmation by quantitative or colorimetric end-tidal carbon dioxide monitoring.^9^,^14^ To examine independent predictors of first-pass success, we fit a logistic regression model with the generalized estimating equations accounting for patient clustering within the EDs. Based on clinical plausibility and a priori knowledge, we chose a set of patient-level variables: age, sex, principal indication for intubation, intubation methods, intubation devices, and intubator’s training level and specialty.^4^,^11^,^15^ Age variables were treated as the categorical variable according to a previous study.^4^ The significance of clinically meaningful interactions was tested as a group to avoid inflating type I error. Specifically, we tested for (intubator’s specialty –×– the use of RSI) interactions using likelihood ratio test; however, preliminary results did not indicate the presence of any effect modifications (data not shown). In the sensitivity analysis, we repeated the analysis in children <10 years based on the literature.^4^ Analysis was conducted with JMP version 10.0.2 (SAS Institute Inc., Cary, NC) and R version 3.1.3 (R Development Core Team, Vienna, Austria).^16^ We considered two-sided P<0.05 statistically significant.

## RESULTS

Of the 8,192 patients who underwent emergency airway management during the study period, 7,786 were recorded in the studies (capture rate, 96%). Of these, 300 were children. After excluding seven children who underwent cricothyroidotomy, tracheostomy, or nasal intubation, the remaining 293 children were eligible for the current study.

Overall, the median age of children was six years (IQR, 1–15 years), and 43% were female (Table 1). The intubations for medical indications accounted for two-thirds of intubations. Cardiac arrest (both medical and traumatic arrests) accounted for approximately 30%. Rapid sequence intubation (RSI) was used in approximately one-fourth of children. Direct laryngoscope was used in more than 90% of the first intubation attempts. EPs (including emergency medicine residents) performed approximately 40% of the first intubation attempts. The overall success rate on first intubation attempt was 60% (95% CI, 54%–66%); success rate was lowest in children aged <2 years (50%; 95% CI, 40%–60%).

Table 2 summarizes the results of multivariable analysis. Children aged 10–18 years (adjusted odds ratio [aOR], 2.45; 95% CI, 1.23–4.87), use of RSI (aOR, 2.17; 95% CI, 1.31–3.57), and intubation attempt by an EP (aOR, 3.21; 95% CI, 1.78–5.83) or by another specialty (adjusted OR, 2.63; 95% CI, 1.51–4.55) were significantly associated with a higher chance of first-pass success. In the sensitivity analysis limiting to children aged <10 years, use of RSI (aOR, 3.05; 95% CI, 1.63–5.70) and intubation attempt by an EP (aOR, 4.08; 95% CI, 1.92–8.63) were also significant predictors of first-pass success.

## DISCUSSION

In this analysis based on two multicenter prospective studies of ED airway management, we found that the first-pass success rate in children was 60%. We also found that older age, use of RSI, and intubator’s training level (beyond transitional year post-graduate year 1 or 2 resident) and specialty (e.g., EPs) were significant predictors of first-pass success in pediatric intubation. To our knowledge, this is the largest study to have examined children who underwent intubation in the ED. Our data build on previous smaller reports about the predictors of intubation success, a finding of clinical and research importance.

To date, a body of evidence supports the importance of first-pass success of intubation in the ED.^1^–^4^ Multiple intubation attempts were associated with increased rate of adverse events.^2^ These adverse events may be due to children’s limited cardiovascular and pulmonary reserves, which result in a limited ability to tolerate extended periods without proper ventilation and oxygenation.^3^ Therefore, investigating factors associated with first-pass success becomes critical for children who require emergency airway management in the ED. There have been few studies that evaluated the predictors of first-pass success among children in the ED – older age, the use of RSI, and intubator’s specialty were suggested as predictors.

In the present study, the older age was associated with a higher odds of first-pass success, consistent with previous studies.^5^,^17^ We surmise that this finding is related to the increasingly adult-like airway anatomy as children age. In addition, EPs might be less familiar with the intubation for pediatric populations because pediatric ED intubation is an infrequent procedure.^18^

A multicenter study from South Korea (n=281) found no association between RSI use and first-pass success rates.^4^ By contrast, a multicenter prospective study from North America (n=156) reported a significant association of RSI use with a higher first-pass success in the ED.^5^ This discrepancy might be attributable to differences in the study design, patient population, setting, training systems, or any combination of these factors. Our multicenter prospective data with the largest sample size and a high capture rate corroborate these findings and extend them by demonstrating the robustness of the associations between the use of RSI and high first-pass success in the ED. The rate of RSI use in the current study, however, was relatively lower compared to those in the previous findings^12^,^16^ because there was a high degree of variation in airway management practices among the EDs in Japan (e.g., the proportion of RSI intubation in non-cardiac-arrest patients ranging from 0% to 79% across the EDs).^9^ Nevertheless, to address this concern, we constructed a logistic regression model with the generalized estimating equations accounting for patient clustering within the EDs.

The use of video laryngoscope had a lower odds of first-pass success but was not significant in this study. This result was not consistent with the literature showing the superior effectiveness of RSI and video laryngoscope in the emergency airway management.^19^,^20^ The potential explanation includes the presence of residual confounders between these factors and first-pass success (e.g., individual training level), the limited statistical power owing to the small number of video laryngoscope use, the use of video laryngoscope by novices, and random errors.

We also found that the intubator’s training level and specialty was significantly associated with first-pass success. This finding is consistent with the South Korean study that reported intubator’s specialty (i.e., EP) had an independent effect on a higher chance of first-pass success.^4^ Emergency airway management in children should ideally be performed by a well-trained intubator in the ED. However, studies have reported that resident physicians have neither sufficient opportunity nor training with close supervision to become proficient at intubations because pediatric intubation is less-frequent procedure.^21^,^22^ Attempting to provide the best possible care to children with limited resources in a system is a challenge. The current literature proposes solutions to improve intubation success through the use of video laryngoscope^23^ and enhanced training (e.g., simulation-based curricula and supplemental operating room training).^24^ However, these measures address only isolated aspects of the critical procedure. Our findings not only facilitate studies to identify optimal airway management strategies but also underscore the importance of continued efforts to improve the quality of emergency airway management in critically-ill children in the ED.

## LIMITATIONS

The current study has several potential limitations. First, our data were subject to self-reporting bias. However, we used the previously applied standardized data collection system with uniform definitions and high capture rates.^9^,^14^ Second, as with any observational study, the observed association might be confounded by unmeasured factors, such as underlying comorbidities and difficulty in intubation. Another potential confounding factor is individual intubator’s skillset in airway management; however, we did adjust for intubator’s level of training and specialty to help account for this possible confounder. Finally, our sample consisted predominantly of academic EDs in Japan; therefore, our inferences may not be generalizable to the other healthcare settings.

## CONCLUSION

In summary, on the basis of two multicenter prospective studies of children who underwent emergency airway management in the ED, we found that older age, use of RSI, and intubator’s training level and specialty were the independent predictors of first-pass success. Given the relatively lower rates of the use of RSI and video laryngoscope, our results encourage the use of RSI with standardized intubation protocol for pediatric emergency intubation and further investigations for first-pass success (e.g., the use of video laryngoscope). In addition, our findings should facilitate further investigation to improve the ability of clinicians to predict the intubation success and continued efforts to improve training systems, which will, in turn, lead to better outcomes in critically-ill children in the ED.

## Figures and Tables

**Table 1:** Characteristics and airway management of 293 pediatric patients receiving intubation in the emergency department.

Variables	Overall (n=293)	Age <2 years (n=94)	Age 2–9 years (n=87)	Age 10–18 years (n=112)
Patient characteristics				
Age, median (IQR), y	6 (1–15)	0 (0–1)	5 (3–7)	16 (13–17)
Weight, median (IQR), kg	20 (10–50)	7 (4–10)	18 (15–23)	45 (50–60)
Female sex	127 (43%)	46 (49%)	36 (41%)	45 (40%)
Primary indication				
Cardiac arrest	86 (29%)	29 (31%)	22 (25%)	35 (31%)
Medical encounters	138 (47%)	60 (64%)	39 (45%)	39 (35%)
Trauma encounters	69 (24%)	5 (5%)	26 (30%)	38 (34%)
Airway management				
Methods				
Rapid sequence intubation	76 (26%)	21 (22%)	19 (22%)	36 (32%)
Sedation without paralytics	70 (24%)	19 (20%)	28 (32%)	23 (21%)
No medication	134 (48%)	50 (53%)	35 (40%)	49 (44%)
Other*	13 (4%)	4 (4%)	5 (6%)	4 (4%)
Devices				
Direct laryngoscope	276 (94%)	94 (100%)	83 (95%)	99 (88%)
Video laryngoscope	12 (4%)	0 (0%)	3 (3%)	9 (8%)
Others†	5 (2%)	0 (0%)	1 (1%)	4 (4%)
Specialty				
Transitional year resident‡	53 (18%)	16 (17%)	10 (11%)	27 (24%)
Emergency physician§	127 (43%)	21 (22%)	39 (45%)	67 (60%)
Pediatrician	45 (15%)	28 (30%)	14 (16%)	3 (3%)
Other specialty||	68 (23%)	29 (31%)	24 (28%)	15 (13%)
Number of intubation attempts until success				
1	176 (60%)	47 (50%)	51 (59%)	78 (69%)
2	69 (24%)	26 (28%)	20 (23%)	23 (21%)
3 or more	47 (16%)	20 (21%)	16 (18%)	11 (10%)
Unknown¶	1 (1%)	1 (1%)	0 (0%)	0 (0%)

*IQR*, interquartile range.

Data were expressed as n (%) unless otherwise indicated.

Percentages may not equal 100 due to rounding.

* Defined as intubation using topical anesthesia or paralytics without sedatives.

† Defined as flexible bronchoscope, or a combination of a gum elastic bougie with direct laryngoscope or video laryngoscope.

‡ Defined as post graduate years 1 or 2.

§ Including emergency medicine residents and emergency attending physicians.

|| Including anesthesiologists and surgeons.

¶ The first intubation attempt was performed and failed in the emergency department, and the subsequent intubation attempts were performed in the operating room.

**Table 2:** Multivariable predictor of first-pass success of emergency intubation in pediatric patients.

	Primary model		Sensitivity analysis (age <10 years)	
	
Variables	Adjusted odds ratio (95% CI)	P value	Adjusted odds ratio (95% CI)	P-value
Age				
Age <2 years	[reference]	--	[reference]	--
Age 2–9 years	1.45 (0.74–2.84)	0.28	--	--
Age10–18 years	2.45 (1.23–4.87)	0.01	--	--
Age 2–7 years	--	--	1.38 (0.66–2.88)	0.39
Age 8–9 years	--	--	1.52 (0.47–4.89)	0.48
Sex				
Male	[reference]	--	[reference]	--
Female	0.64 (0.41–1.01)	0.05	0.69 (0.38–1.24)	0.21
Primary indication				
Cardiac arrest	[reference]	--	[reference]	--
Medical	0.56 (0.30–1.05)	0.07	0.61 (0.28–1.33)	0.21
Trauma	0.64 (0.29–1.40)	0.26	0.49 (0.17–1.45)	0.20
Methods				
non-RSI	[reference]	--	[reference]	--
RSI	2.17 (1.31–3.57)	0.002	3.05 (1.63–5.70)	<0.001
Devices				
Direct laryngoscope	[reference]		--¶	--
Video laryngoscope	0.31 (0.08–1.23)	0.10	--¶	--
Others*	0.24 (0.03–2.08)	0.20	--¶	--
Specialty				
Transitional year resident†	[reference]	--	[reference]	--
Emergency physician‡	3.21 (1.78–5.83)	<0.001	4.08 (1.92–8.63)	<0.001
Pediatrician	2.07 (0.96–4.47)	0.06	2.36 (1.11–4.97)	0.03
Other specialty§	2.63 (1.51–4.55)	0.001	2.39 (1.32–4.32)	0.004

*CI*, confidence intervals; *RSI*, rapid sequence intubation.

* Defined as flexible bronchoscope, or a combination of a gum elastic bougie with direct laryngoscope or video laryngoscope.

† Defined as post-graduate-years 1 or 2.

‡ Including emergency medicine residents and emergency attending physicians.

§ Including anesthesiologists and surgeons.

¶ Excluded the intubation device variable from the multivariable analysis as only 4 intubation attempts were performed with video laryngoscope or other device in children aged <10 years.
